# Dual Infection with *Ehrlichia chaffeensis* and a Spotted Fever Group Rickettsia: A Case Report—Reply to Dr. Sulzer

**DOI:** 10.3201/eid0404.980431

**Published:** 1998

**Authors:** Daniel J. Sexton, David H. Walker

**Affiliations:** Duke University Medical Center, Durham, North Carolina, USA; and University of Texas Medical Branch at Galveston, Galveston, Texas, USA


### Dual Infection with *Ehrlichia chaffeensis* and a Spotted Fever Group Rickettsia: A Case Report—Reply to Dr. Sulzer

**To the Editor:** Several investigators have suggested that some of Wilson and Chowning's patients may have had coinfection with *Babesia* and *Rickettsia rickettsii* (1-4). Furthermore, the organisms that Wilson and Chowning observed in red cells of 20% of the local Columbian ground squirrels are consistent with later reports of various species of *Babesia* in the erythrocytes of other species of squirrels (4). However, most rickettsiologists who have commented on Wilson and Chowning's paper have concluded that intraerythrocytic organisms observed in blood samples did not contribute substantially to the illnesses of the 23 patients described. Although Stiles, Wenyon, and Brumpt concluded that the organisms in human blood samples observed by Wilson and Chowning were artifacts or malarial parasites (5-7), contemporary experts who have reviewed the colored plates that accompanied Wilson and Chowning's 1904 paper believe that there is "little" or "no doubt" that Wilson and Chowning actually described organisms of the genus *Babesia* (1,2,8).

In a commentary that followed the republication of Wilson and Chowning's landmark paper in 1979 (9), Richard Ormsbee reviewed the sequence of events that followed the publication of Wilson and Chowning's report in 1904 (10). After more than 200 hours of careful microscopy, C.W. Stiles could find no evidence of *Pyroplasma* in the blood of 12 patients with Rocky Mountain spotted fever (RMSF). He refuted Wilson and Chowning's findings (5) and challenged Chowning, who was also in the Bitter Root Valley, to demonstrate the presence of organisms in the blood of a person with a typical case of RMSF. Chowning was unable to find *Pyroplasma* in blood smears from these patients (10). Ricketts did not arrive in the Bitter Root Valley to begin his studies of RMSF until 1906 (11); thus he could not have published his classic paper on the etiology of RMSF in volume 1 of the Journal of Infectious Diseases.

To our knowledge, ecologic studies done in the Bitter Root Valley have not demonstrated endemic foci of babesial infection. A serologic survey of 246 Bitter Root Valley residents in 1978 showed no antibabesial antibodies (12). Although it is possible that 4 of the 23 patients with RMSF described by Wilson and Chowning had incidental preexisting latent babesial infection, the clinical and autopsy data they presented suggest that the patients had typical *R. rickettsii* infection. There is no proof that any of the patients described by Wilson and Chowning had simultaneous acute babesial and rickettsial infection, and we agree with Ormsbee that the significance of the "*Pyroplasma hominis*" described in the blood smears of several of Wilson and Chowning's patients is "... a mystery that persists to this day" (10).

**Daniel J. Sexton* and David H. Walker†**
Duke University Medical Center, Durham, North Carolina, USA; and University of Texas Medical Branch at Galveston, Galveston, Texas, USA

### References

1. Gorenflot A, Mourbri K, Precigout E, Carcy B. Human babesiosis. Ann Trop Med Paristol 1998;92:489-501.
2. Hoare CA. Comparative aspects of human babesiosis. Trans R Soc Trop Med Hyg 1980;74:143-8.
3. Ruebush TK II, Cassaday PB, Marsh HJ, Lisker SA, Voorhers D, Mahoney EB, et al. Human babesiosis on Nantucket Island. Clinical features. Ann Intern Med 1977;86:6-9.
4. Pinsky RL, Lutwick LI. Spotted fever and babesiosis. Rev Infect Dis 1979:1:895.
5. Stiles CW. A zoological investigation into the cause, transmission, and source of Rocky Mountain "spotted fever." Hygienic Laboratory Bulletin . Washington: Government Printing Office; 1905. Publication No 20:7-119.
6. Wenyon CM. Protozoology. Vol 2. London: W. Wood; 1926.
7. Brumpt E. Precis de parasitologie. 5th ed. Paris: Masson et Cie; 1936. p. 497.
8. Pruthi RK, Marshall WF, Wiltsie JC, Persing DH. Human babesiosis. Mayo Clin Proc 1995:70:853-62.
9. Wilson LB, Chowning WM. Studies in Pyroplasmosis hominis ('spotted fever" or "tick fever of the Rocky Mountains). Rev Infect Dis 1979;1:540-58.
10. Ormsbee RA. Studies in Pyroplasmosis hominis ("spotted fever" or "tick fever" of the Rocky Mountains) by Louis B. Wilson and William A. Chowning. Rev Infect Dis 1979;1:559-62.
11. Harden VA. Rocky Mountain spotted fever research and the development of the insect vector theory, 1900-1930. Bull Hist Med 1985;59:449-66.
12. Chisholm ES, Ruebush TK II, Sulzer AJ, Healy GR. *Babesia microti* infection in man: evaluation of an indirect immunofluorescent antibody test. Am J Trop Med Hyg 1978:27:14-9.

**To the Editor:** Several investigators have suggested that some of Wilson and Chowning's patients may have had coinfection with *Babesia* and *Rickettsia rickettsii* (1-4). Furthermore, the organisms that Wilson and Chowning observed in red cells of 20% of the local Columbian ground squirrels are consistent with later reports of various species of *Babesia* in the erythrocytes of other species of squirrels (4). However, most rickettsiologists who have commented on Wilson and Chowning's paper have concluded that intraerythrocytic organisms observed in blood samples did not contribute substantially to the illnesses of the 23 patients described. Although Stiles, Wenyon, and Brumpt concluded that the organisms in human blood samples observed by Wilson and Chowning were artifacts or malarial parasites (5-7), contemporary experts who have reviewed the colored plates that accompanied Wilson and Chowning's 1904 paper believe that there is "little" or "no doubt" that Wilson and Chowning actually described organisms of the genus *Babesia* (1,2,8).

In a commentary that followed the republication of Wilson and Chowning's landmark paper in 1979 (9), Richard Ormsbee reviewed the sequence of events that followed the publication of Wilson and Chowning's report in 1904 (10). After more than 200 hours of careful microscopy, C.W. Stiles could find no evidence of *Pyroplasma* in the blood of 12 patients with Rocky Mountain spotted fever (RMSF). He refuted Wilson and Chowning's findings (5) and challenged Chowning, who was also in the Bitter Root Valley, to demonstrate the presence of organisms in the blood of a person with a typical case of RMSF. Chowning was unable to find *Pyroplasma* in blood smears from these patients (10). Ricketts did not arrive in the Bitter Root Valley to begin his studies of RMSF until 1906 (11); thus he could not have published his classic paper on the etiology of RMSF in volume 1 of the Journal of Infectious Diseases.

To our knowledge, ecologic studies done in the Bitter Root Valley have not demonstrated endemic foci of babesial infection. A serologic survey of 246 Bitter Root Valley residents in 1978 showed no antibabesial antibodies (12). Although it is possible that 4 of the 23 patients with RMSF described by Wilson and Chowning had incidental preexisting latent babesial infection, the clinical and autopsy data they presented suggest that the patients had typical *R. rickettsii* infection. There is no proof that any of the patients described by Wilson and Chowning had simultaneous acute babesial and rickettsial infection, and we agree with Ormsbee that the significance of the "*Pyroplasma hominis*" described in the blood smears of several of Wilson and Chowning's patients is "... a mystery that persists to this day" (10).
